# Rabbit model of *Staphylococcus aureus* implant-associated spinal infection

**DOI:** 10.1242/dmm.045385

**Published:** 2020-07-28

**Authors:** Oren Gordon, Robert J. Miller, John M. Thompson, Alvaro A. Ordonez, Mariah H. Klunk, Dustin A. Dikeman, Daniel P. Joyce, Camilo A. Ruiz-Bedoya, Lloyd S. Miller, Sanjay K. Jain

**Affiliations:** 1 Division of Infectious Diseases, Department of Pediatrics, Johns Hopkins University School of Medicine, Baltimore, MD 21287, USA; 2 Center for Infection and Inflammation Imaging Research, Johns Hopkins University School of Medicine, Baltimore, MD 21287, USA; 3 Department of Dermatology, Johns Hopkins University School of Medicine, Baltimore, MD 21287, USA; 4 Department of Orthopedic Surgery, Johns Hopkins University School of Medicine, Baltimore, MD 21287, USA; 5 Immunology, Janssen Research and Development, Spring House, PA 19477, USA

**Keywords:** Spinal infection, Post-surgical infection, Implant-associated infection, Bioluminescence, PET

## Abstract

Post-surgical implant-associated spinal infection is a devastating complication commonly caused by *Staphylococcus aureus*. Biofilm formation is thought to reduce penetration of antibiotics and immune cells, contributing to chronic and difficult-to-treat infections. A rabbit model of a posterior-approach spinal surgery was created, in which bilateral titanium pedicle screws were interconnected by a plate at the level of lumbar vertebra L6 and inoculated with a methicillin-resistant *S.* *aureus* (MRSA) bioluminescent strain. *In vivo* whole-animal bioluminescence imaging (BLI) and *ex vivo* bacterial cultures demonstrated a peak in bacterial burden by day 14, when wound dehiscence occurred. Structures suggestive of biofilm, visualized by scanning electron microscopy, were evident up to 56 days following infection. Infection-induced inflammation and bone remodeling were also monitored using ^18^F-fluorodeoxyglucose (^18^F-FDG) positron emission tomography (PET) and computed tomography (CT). PET imaging signals were noted in the soft tissue and bone surrounding the implanted materials. CT imaging demonstrated marked bone remodeling and a decrease in dense bone at the infection sites. This rabbit model of implant-associated spinal infection provides a valuable preclinical *in vivo* approach to investigate the pathogenesis of implant-associated spinal infections and to evaluate novel therapeutics.

## Abstract (precis)

**Summary:** A model of post-surgical methicillin-resistant *Staphylococcus aureus* implant-associated spinal infection was created in rabbits, recapitulating acute infection as well as chronic low-burden infection, with structures suggestive of biofilm formation and bone remodeling.

## INTRODUCTION

Post-surgical implant-associated spinal infection (IASI) is one of the most common causes of morbidity following spinal surgery and can potentially lead to neurological sequelae and disability. The reported infection rate following spinal surgery varies between 0.5% and 18.8% and is dependent mostly on patient population and the procedure performed (Beiner et al., 2003; Chahoud et al., 2014; Smith et al., 2011). Patients with a history of trauma, diabetes, advanced age or neuromuscular scoliosis are the groups with the highest risk (Aleissa et al., 2011; McDermott et al., 2012; Smith et al., 2011; Sponseller et al., 2013, 2000). Removal of the implanted materials is often not feasible due to the risk of spine destabilization, necessitating repeated surgical interventions and prolonged antibiotic therapy. Still, there is risk for treatment failure and recurrence in the months or years following infection. In a series of 102 IASI patients, treatment failure occurred in 36%, with a median time to failure of 113 days (Cho et al., 2018). Another study followed 253 patients with vertebral osteomyelitis for a median of 6.5 years and relapse occurred in 14% (McHenry et al., 2002). In some rare cases, recurrence may even occur decades following the initial infection (Mansour et al., 1979). *Staphylococcus aureus* is the most common bacterial pathogen recovered from IASI (Jiménez-Mejías et al., 1999; Pull ter Gunne et al., 2010), and the emergence of methicillin-resistant *S. aureus* (MRSA) poses an increased risk of treatment failure (Cho et al., 2018). Biofilm formation is thought to reduce antibiotic and immune cell penetration, leading to persistent and difficult-to-treat infections (Cho et al., 2018; Sampedro et al., 2010). The diagnosis of IASI is also difficult to establish, but is suggested by the presence of a sinus tract and persistent or late postoperative wound drainage or dehiscence (Chahoud et al., 2014; Jiménez-Mejías et al., 1999). Non-invasive diagnosis of IASI is challenging, and evaluation of progression as well as response to treatment relies on non-specific inflammatory markers in serum (McDermott et al., 2012). ^18^F-fluorodeoxyglucose (^18^F-FDG) positron emission tomography (PET) is a complementary tool often used to image IASI and might also be beneficial to evaluate disease progression (Gemmel et al., 2010).

Animal models for post-surgical IASI provide an *in vivo* approach to study the pathophysiology and to evaluate preventative measures, diagnostic tools and treatment strategies. Current animal models in mice, rats and rabbits use a variety of surgical procedures, which may not fully recapitulate the complex surgical techniques and orthopedic hardware in humans (Guiboux et al., 1998; Laratta et al., 2017a,b; Ofluoglu et al., 2007; Poelstra et al., 2000; Stavrakis et al., 2015). In addition, most of these prior animal models of IASI were evaluated in an acute time period following surgery and infection (i.e. 5-14 days) and there was not an opportunity to evaluate the persistent infection and inflammation that occur in human IASI. Despite being an eminent part of IASI, only one study directly evaluated biofilm formation on the spinal hardware (Hazer et al., 2016). Prior studies have also utilized a mouse model of IASI in conjunction with a bioluminescent *S. aureus* strain and *in vivo* bioluminescence imaging (BLI) (Dworsky et al., 2017). This useful model was employed to evaluate local antibiotic treatment (Hu et al., 2017), implant antimicrobial coating (Hegde et al., 2018) and multimodal imaging for surgical management (Zoller et al., 2019).

In order to better simulate the surgical procedures and implants involved in human IASI, we developed a post-surgical IASI model in rabbits using a surgical approach and orthopedic-grade implants. Using this model, *in vivo* bacterial burden, infection-induced inflammation and bone remodeling were monitored using non-invasive imaging [bioluminescence, PET, computed tomography (CT)] and *ex vivo* analysis to study both acute and chronic disease.

## RESULTS

### Monitoring of bacterial burden by non-invasive *in vivo* imaging and *ex vivo* confirmation

**Fig. 1.**
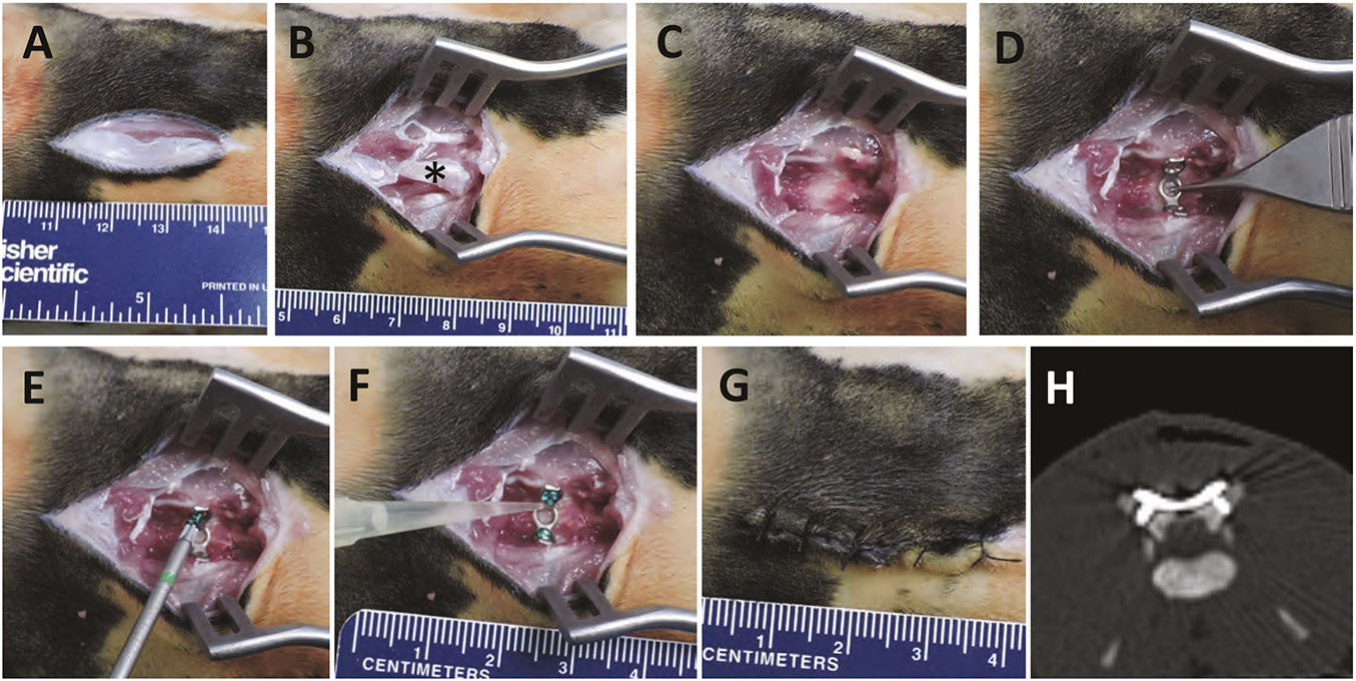
**Rabbit surgical procedures.** (A) A midline 3 cm incision was made with a 15-blade scalpel through the skin at the area of L5-L6. (B) Surgical dissection was directed bilaterally along the L6 spinous process (marked with an asterisk). (C) The L6 spinous process was removed using a small rongeur, creating a hollow self-contained defect, mimicking a partial laminectomy defect. (D) An orthopedic-grade titanium plate (0.6 mm×12 mm with pre-structured screw holes) was placed into the defect and over the L6 transverse processes. (E) Self-drilling pedicle screws (1.5 mm×4 mm) were used to fix the plate. (F) An inoculum of the bioluminescent MRSA strain SAP231 in 100 μl PBS was pipetted onto the implanted screws and plate. (G) The surgical site was closed with absorbable sutures. (H) A representative CT image demonstrating the correct positioning of the screws within the bilateral pedicles.

**Fig. 2.**
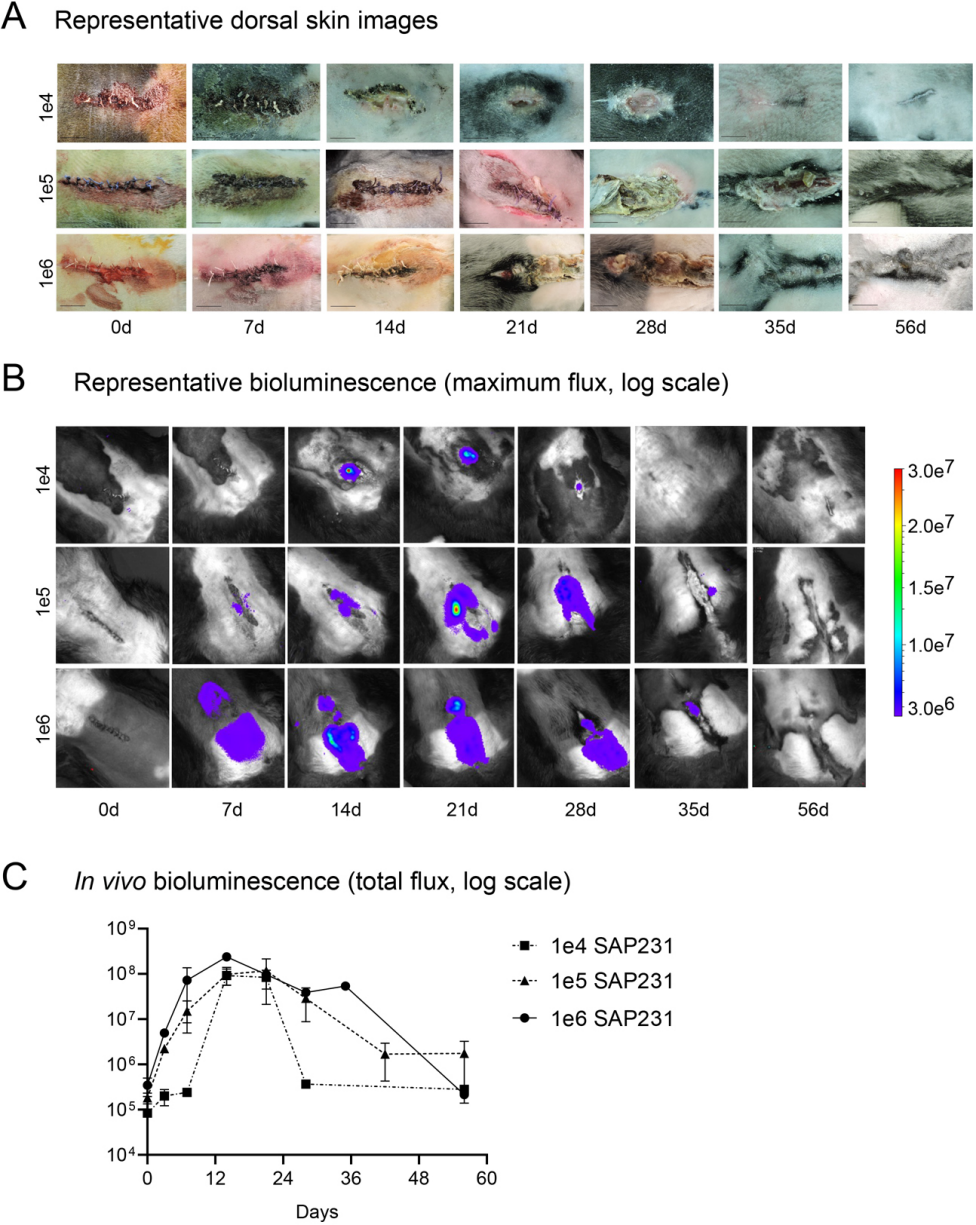
**Longitudinal measurement of bacterial burden with *in vivo* bioluminescence.** The rabbit model of IASI was performed with different inocula of SAP231 [1×10^4^ (*n*=2), 1×10^5^ (*n*=5) or 1×10^6^ (*n*=3) CFUs]. *In vivo* bioluminescence imaging (BLI) was performed on post-operative days 0, 3, 7 and once weekly thereafter for a total of 56 days. (A) Representative dorsal skin images showing the surgical site. Note that wound dehiscence with expression of pus occurred in all rabbits starting at day 14. Scale bars: 1 cm. (B) Representative *in vivo S. aureus* BLI signals on a color scale overlaid on top of a grayscale image of the backs of the rabbits. (C) Mean *in vivo* BLI [total flux (photons/s)±s.e.m. (logarithmic scale)].

**Fig. 3.**
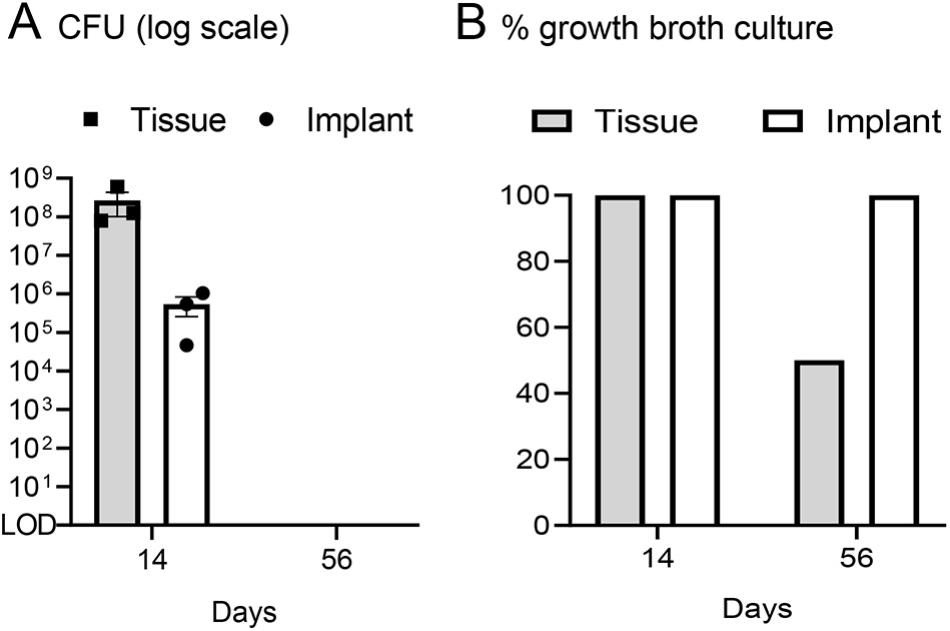
**Confirmation of bacterial burden.** The rabbit model of IASI was performed with 1×10^5^ CFUs of SAP231 (*n*=3 rabbits per time point). On post-operative days 14 and 56, the implants were removed and sonicated, and the infected vertebra with adjacent upper and lower vertebrae and surrounding soft tissue were harvested and homogenized. (A) Mean *ex vivo* CFUs±s.e.m. from tissue and implant specimens. (B) Tissue homogenates and implants were cultured for an additional 48 h in shaking broth cultures followed by overnight culture on plates and the presence or absence of CFUs were determined. Data are presented as the percentage of tissue or implant specimens with CFUs present.

**Fig. 4.**
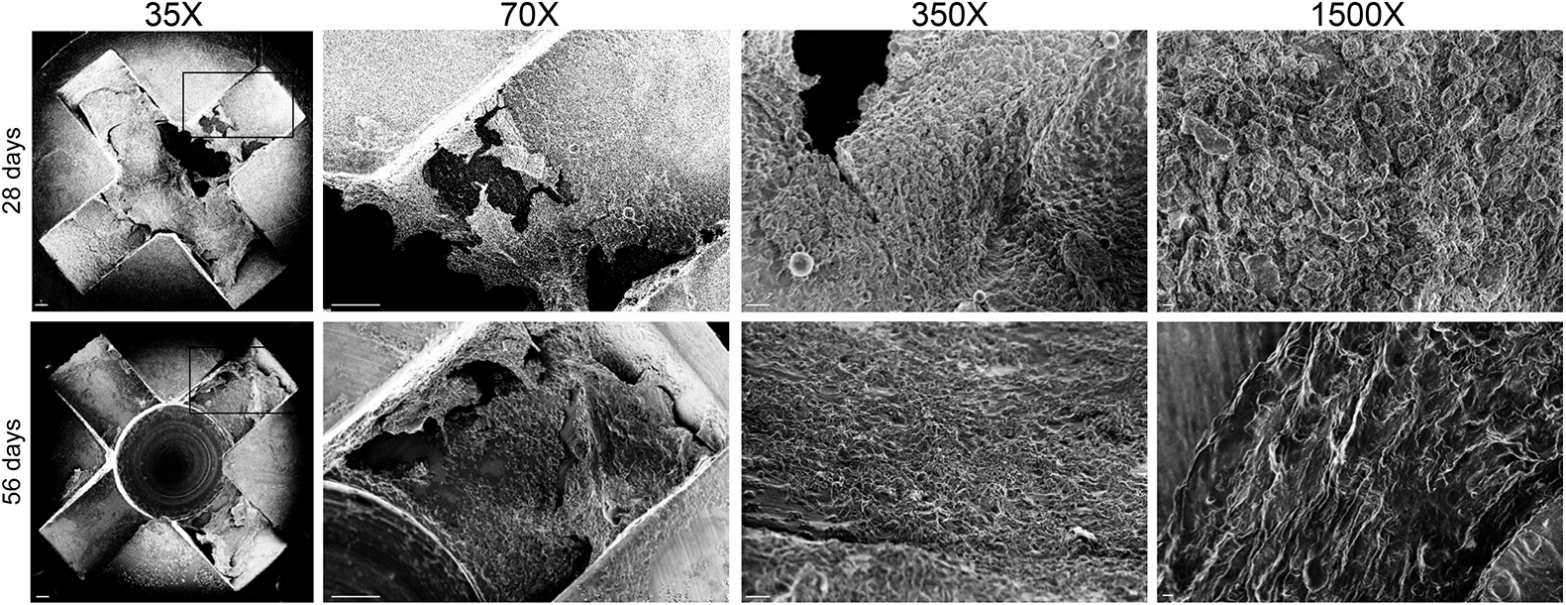
**Bacterial biofilm formation on the *ex vivo* spinal implants.** The rabbit model of IASI was performed with 1×10^5^ CFUs of SAP231 (*n*=3 rabbits per time point). Implants were harvested on post-operative days 28 or 56. Representative SEM images of the head of the pedicle screws are shown. Four magnifications are shown, as indicated. The boxed areas are enlarged in the 70× magnification images. Scale bars (from left to right for upper and lower rows): 100 μm, 100 μm, 20 μm and 2 μm. Data are mean±s.e.m.

The rabbit IASI was performed as described in Fig. 1 with three different inocula [1×10^4^, 1×10^5^ or 1×10^6^ colony-forming units (CFUs)] of the bioluminescent MRSA strain SAP231. Non-invasive *in vivo* whole-animal BLI (on post-operative days 0, 3, 7 and once weekly thereafter for a total of 56 days) demonstrated a peak bacterial burden by day 14, when wound dehiscence also occurred (Fig. 2A-C). Note that for 1×10^4^ CFUs, BLI signal appeared only after wound dehiscence occurred and within the open wound (Fig. 2A,B, top row). For higher inocula, the *in vivo* BLI signals were apparent before wound dehiscence. The BLI signals decreased gradually over the 56-day experiment with all inocula (Fig. 2B,C). By post-operative day 56 there were no *in vivo* BLI signals detected in any of the rabbits and all of the wounds overlying the post-operative sites spontaneously healed (Fig. 2A-C). On post-operative day 14, *ex vivo* CFU enumeration confirmed high burden of infection for soft tissue and bone as well as the hardware (Fig. 3A). In contrast, on post-operative day 56, there were no *ex vivo* CFUs recovered from the tissue or implants. To identify the presence or absence of any remaining bacteria in the tissue specimens or adherent to the implants, soft tissue and bone homogenates as well as implant sonicates were cultured in shaking broth for an additional 48 h followed by detection of CFUs after overnight culture on plates. On post-operative day 14, 100% of the tissue and implant specimens had bacterial CFUs present (Fig. 3B). However, on day 56, only 50% of the tissue specimens and 100% of the implant specimens had bacteria present, and all recovered bacterial colonies were bioluminescent as expected. On post-operative days 28 and 56, structures suggesting biofilm formation with viscous fibers were identified on all of the *ex vivo* screws and plates (Fig. 4), although these structures were less extensive on day 56 compared with day 28. Bacterial biofilms were deemed present if characteristic features such as viscous fibers were present.

### ^18^F-FDG-PET/CT

**Fig. 5.**
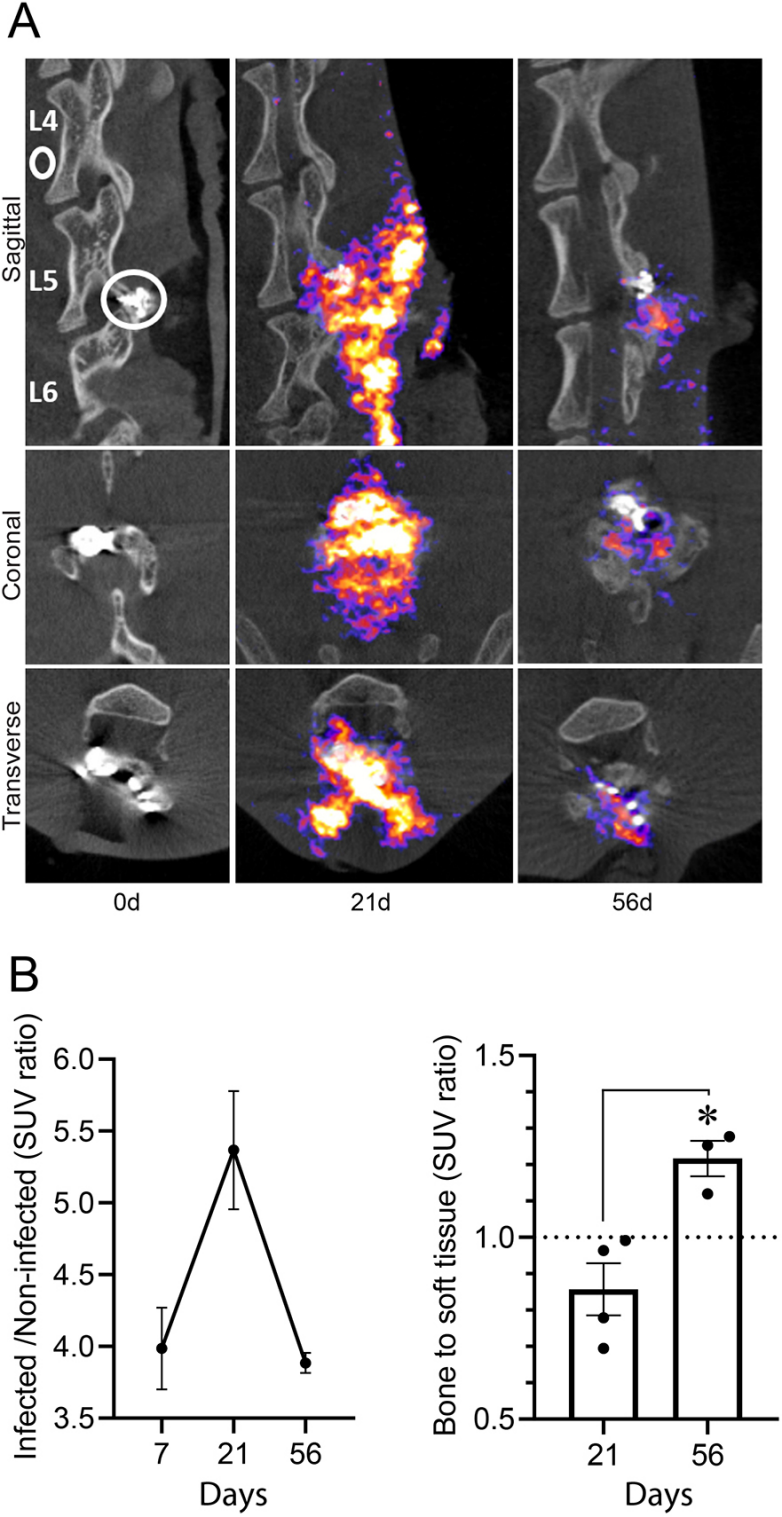
**FDG-PET/CT imaging in early and late time points of the IASI.** The rabbit model of IASI was performed with 1×10^6^ CFUs of SAP231. On post-operative days 7, 21 and 56 (*n*=2-4 rabbits per time point), rabbits were imaged with ^18^F-FDG-PET/CT imaging. (A) Representative images of ^18^F-FDG-PET/CT (left column shows CT images only). Representative regions of interest (ROIs) are noted around the implant (infected) and anteriorly to L4 (non-infected reference). (B) Standard uptake values (SUVs) are presented as ratios between infected and non-infected ROIs (left) or bone-to-soft tissue SUV ratios (right). *P=0.03 (two-tailed Mann–Whitney test). Data are mean±s.e.m.

On post-operative days 7, 21 and 56, ^18^F-FDG-PET/CT imaging was performed (Fig. 5). The ^18^F-FDG-PET imaging demonstrated distinct and localized uptake at the site of infection (Fig. 5A). In agreement with *in vivo* BLI signals (Fig. 2B,C), the standard uptake value (SUV) ratios of the ^18^F-FDG-PET imaging signals between the infected site surrounding the hardware and a non-infected reference increased from 3.99±0.23 on post-operative day 7 to 5.37±0.41 on post-operative day 21 (Fig. 5B, left). ^18^F-FDG uptake decreased by day 56 (SUV ratio=3.88±0.04), but the ^18^F-FDG uptake signals were still visible and localized to the site of infection, likely indicating persistent inflammation. During the 56-day course of infection, the ^18^F-FDG-PET signal from bone did not statistically change, with bone-to-non-infected reference SUV ratios of 2.55±0.14, 2.30±0.07 and 2.21±0.13 on post-operative days 7, 21 and 56, respectively. However, bone-to-soft tissue ratios had statistically significant changes as they were 0.86±0.12 and 1.22±0.07 on post-operative days 21 and 56, respectively (*P*<0.05) (Fig. 5B, right), suggesting an increase in bone inflammation at the later time point.

### Infection-induced changes in bone

**Fig. 6.**
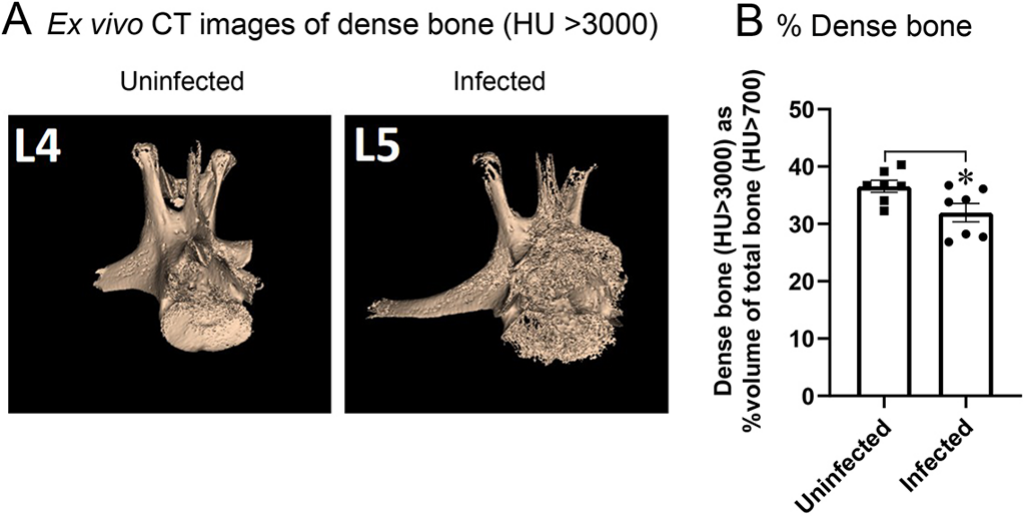
**Bone changes after the post-surgical IASI model by *ex vivo* CT imaging.** The rabbit model of IASI was performed with 1×10^6^ CFUs of SAP231 (*n*=3). At post-operative day 56, rabbits were euthanized and the implants removed to avoid metal-related imaging artifacts. Infected (L5-L6) and surrounding vertebrae (L4) were imaged by *ex vivo* CT. (A) Representative CT images of bone in uninfected (L4) or infected (L5) vertebrae. (B) The percentage of dense bone volume (HU>3000) divided by the total bone volume (HU>700) are presented. *P=0.04 (two-tailed Mann–Whitney test). Data are mean±s.e.m.

On post-operative day 56, rabbits were euthanized and infection-induced changes in the bone were evaluated by high-resolution *ex vivo* CT imaging. Visualization of lumbar vertebrae showed that the infected vertebra had less dense bone than uninfected vertebra (Fig. 6A). The infected vertebra had decreased amount of dense bone (31.9±1.5%), compared with the uninfected vertebra (36.5±1.0%; *P*<0.05) (Fig. 6B). It should be noted that even small changes in bone density (as low as 2-3%) are clinically significant (Bonnick, 2008).

## DISCUSSION

Post-surgical IASI is a devastating complication of spinal surgical procedures with potentially long-term sequelae. Accurate diagnosis is crucial, and precision medicine tools are being developed for prognostication and risk stratification of patients. In this study, a rabbit model of a post-surgical MRSA IASI was created to more closely simulate the surgical technique and orthopedic-grade hardware used in spinal implant surgery in humans. This model more accurately recapitulates initial infection that often involves wound dehiscence at the post-surgical site. Even though the soft tissue infection overlying the surgical site resolved in all of the rabbits (irrespective of the bacterial inoculum of 1×10^4^, 1×10^5^ or 1×10^6^ CFUs) over a few weeks, bacteria could still be recovered from tissue/bone and implant specimens with scanning electron microscopy (SEM), suggesting biofilm formation on the implants. At the same time, bacteria could not be imaged by bioluminescence. Although BLI is a powerful tool to monitor infection and can image as low as 500 CFUs (Bernthal et al., 2010), in this late, low-level infection, bacterial load was minimal and below the limit of detection for BLI. This indicates that the surgical site had ongoing bacterial persistence, which is representative of the clinical scenario whereby the initial IASI might seem to resolve clinically but can later relapse as a chronic infection, owing to the presence of bacteria adherent to the implanted materials (Cho et al., 2018). Taken together, these results establish a biphasic rabbit model of IASI: (1) an early purulent post-surgical soft-tissue infection with wound dehiscence, and (2) a chronic and persistent infection with bone remodeling. Small changes in bone density (as low as 2-3%) are clinically significant and may modify the risk for complication, such as pathological fractures (Bonnick, 2008). In the current study, there was a 4.6% reduction in bone density (measured by CT) in infected versus uninfected vertebrae, further supporting the translational relevance of this model.

In this model, longitudinal imaging with ^18^F-FDG was able to monitor the infection-induced inflammation as well as localize the infection (i.e. soft tissue versus bone involvement). In recent years, ^18^F-FDG-PET/CT is being increasingly utilized for imaging of infectious diseases (Censullo and Vijayan, 2017). In general, for detection of a vertebral infection, ^18^F-FDG-PET/CT imaging has a relatively high sensitivity (Gemmel et al., 2010; Kouijzer et al., 2018). However, since ^18^F-FDG is a marker for glucose uptake, any metabolically active tissue is detected and this tracer is therefore non-specific and cannot accurately distinguish between inflammation due to infection versus that caused by non-infectious processes. In the future, novel PET-based imaging tracers specific to the pathogen or components of the inflammatory response may serve as precision medicine tools to facilitate acquisition of temporal and spatial information on infection progression, unachievable by current modalities (Censullo and Vijayan, 2017; Gordon et al., 2019; Hammoud, 2016). The extent and location of infection and inflammatory response could facilitate risk stratification of patients with post-surgical IASI. This is crucial for medical decision making involving possible invasive procedures (surgical removal of infected implanted materials, debridement, etc.), and to direct prolonged parenteral or oral antibiotic therapy (Davis et al., 2009; Lin et al., 2016). As PET-based imaging approaches can be easily translated to clinical use (Ordonez et al., 2019), future applications of this technology may contribute valuable information, including delineating bone involvement and detecting persistence of infection.

This study has some limitations. The study was a proof of concept and group sizes were not determined by power calculations. Evaluation of biofilm formation was performed by SEM. This method is limited because it cannot definitely identify bacteria or components of the extracellular matrix and may, in fact, reflect adherent bacteria. Finally, bone remodeling and changes in bone density can be a result of surgery and/or implants rather than infection alone, but this was not addressed in the current study.

Taken together, this study establishes a rabbit model of IASI in which the surgical approaches and orthopedic-grade implants are used to more closely simulate human IASI. By using advanced *in vivo* BLI and PET/CT imaging, the *in vivo* bacterial burden, infection-induced inflammation and bone remodeling could also be measured at acute and chronic time points. This rabbit model could serve a valuable preclinical model of IASI to study the pathogenesis and novel diagnostic and therapeutic modalities prior to studies in larger animals and humans.

## MATERIALS AND METHODS

### Bacteria

The bioluminescent USA300 community-acquired MRSA strain SAP231 was previously derived from the clinical isolate NRS384 (Plaut et al., 2013). SAP231 was previously used in preclinical mouse models, including a prosthetic joint infection model (Bernthal et al., 2010; Pribaz et al., 2012). SAP231 possesses a stable bioluminescent construct integrated into the bacterial chromosome that is maintained in all progeny without selection. Only live and actively metabolizing SAP231 bacteria emit light. SAP231 were streaked onto plates containing tryptic soy broth and Bacto agar (1.5%) (BD Biosciences). Colonies of SAP231 were grown overnight at 37°C in a shaking incubator (240 rpm) in tryptic soy broth. Mid-logarithmic-phase bacteria were obtained after a 2-h subculture of a 1:50 dilution of the overnight culture.

### Rabbits

All procedures were approved by the Johns Hopkins Animal Care and Use Committee. A total of fourteen 10- to 16-week-old male Dutch Belted Rabbits (Robinson Services) were used. All rabbits were housed under specific pathogen-free conditions at an American Association for the Accreditation of Laboratory Animal Care (AAALAC)-accredited animal facility at Johns Hopkins University and were used according to procedures described in the Guide for the Care and Use of Laboratory Animals [National Research Council (US) Committee for the Update of the Guide for the Care and Use of Laboratory Animals, 2011].

Of the 14 rabbits used in this study, two were inoculated with 1×10^4^ CFUs and followed by BLI up to day 56 (Fig. 2). Nine rabbits were inoculated with 1×10^5^ CFUs. Of these nine rabbits, three were sacrificed at day 14 for *ex vivo* CFU enumeration (Fig. 3). Three were sacrificed at day 28 for SEM analysis (Fig. 4) and three were sacrificed at day 56 for CFU enumeration followed by SEM (Figs 3 and 4). Three more rabbits were inoculated with 1×10^6^ CFUs and sacrificed at day 56 for *ex vivo* CT imaging (Fig. 6). All animals were followed with BLI and two to four rabbits from each inocula group were subjected to ^18^F-FDG-PET/CT on post-operative days 7, 21 and 56.

### Surgical procedures

Rabbits were initially anesthetized with intramuscular ketamine (20-30 mg/kg) and xylazine (1-2 mg/kg) and general anesthesia was subsequently maintained with inhalation isoflurane (∼1.5%). Sterile ophthalmic ointment (Rugby, Livonia, MI, USA) was applied to the eyes. For analgesia, sustained-release buprenorphine (0.2 mg/kg) and meloxicam (ZooPharm^®^; 0.6 mg) were administered subcutaneously prior to surgery. The surgical procedure is described in Fig. 1. The overlying back skin was shaved and prepped with isopropyl alcohol (70%) followed by povidone-iodine (10%), each applied thrice. A longitudinal incision was made with a 15-blade scalpel in the midline, ∼3 cm in length, over the lower back at the level of the fifth and sixth lumbar vertebrae (L5-L6). An incision of the fascia was then performed in order to expose the L6 vertebra. Using a small rongeur, the entire spinous process, with surrounding musculature and ligaments, was excised from the base, creating a hollow self-contained defect, mimicking a partial laminectomy defect. Orthopedic-grade self-drilling pedicle screws [1.5 mm (width)×4 mm (length)] were used to fix an orthopedic-grade compatible plate (0.6 mm width) between the two transverse processes of the L6 vertebra (both Zimmer Biomet). The surfaces of the screws and plate were then inoculated with either 1×10^4^, 1×10^5^ or 1×10^6^ CFUs of SAP231 in 100 μl PBS. The surgical site and overlying skin was closed with interrupted 3-0 Vicryl sutures (Ethicon). CT imaging was obtained following surgery to validate the correct location of the implants before continuing further experiments.

### *In vivo* BLI

*In vivo* BLI was performed to non-invasively monitor the bacterial burden longitudinally over time using a Lumina III IVIS system (PerkinElmer, Hopkinton, MA, USA). Rabbits were imaged on post-operative days 0, 3, 7 and once weekly thereafter for a total of 56 days. Bioluminescent signals were overlaid on a grayscale image of the backs of the rabbits and quantified by using total flux (photons/s) within an oval 6×8 cm region of interest (ROI) using Living Image software (PerkinElmer).

### Quantification of *ex vivo* CFUs

Rabbits were euthanized on days 14 and 56 for *ex vivo* CFU quantification of bacterial burden. The screws and plate were removed, and adherent bacteria were isolated by sonication in 0.3% TWEEN solution (Sigma-Aldrich) for 10 min followed by vortexing for 2 min, as previously described (Miller et al., 2019). The infected vertebra with adjacent upper and lower vertebrae and surrounding soft tissue were also harvested and homogenized in a blender container with 200 ml PBS, as previously described (Miller et al., 2019). The CFUs were counted after overnight culture with serial dilutions on plates. To further identify any bacteria remaining in the bone and soft tissue or the implants, tissue homogenates and implant sonicates were cultured for an additional 48 h in shaking tryptic soy broth (240 rpm at 37°C) and the presence or absence of CFUs were determined after overnight culture on plates.

### SEM

Rabbits were euthanized on post-operative days 28 and 56 for SEM, as previously described (Pribaz et al., 2012). Briefly, the *ex vivo* implants were carefully extracted as not to disturb the surface structures and were immediately fixed in buffered formaldehyde (4%) and glutaraldehyde (2.5%) for 48 h and rinsed with buffer thrice (10 min each). Secondary fixation was performed for 1 h with osmium tetroxide (1%) in PBS. Dehydration was carried out by incubating the implants (15 min each) in increasing concentrations of ethanol (30%, 50%, 67%, 80%, 90%, 100% and 100%). Subsequently, the implants were immersed in decreasing mixtures of ethanol and hexamethyldisilazane (HMDS) (ethanol:HMDS=3:1, 1:1 and 1:3) followed by 100% HMDS dehydration twice (15 min each). Samples were air-dried overnight to ensure evaporation of HMDS. All implants were mounted on aluminum stub mounts, sputter-coated with gold-palladium prior to imaging under a field-emission scanning electron microscope (JSM-6700F FE-SEM; JEOL, Tokyo, Japan) at 35×, 70×, 350× and 1500× magnification.

### PET-CT imaging with ^18^F-FDG and image analysis

On post-operative days 7, 21 and 56, rabbits were placed under anesthesia, weighed and 14.04±1.7 MBq ^18^F-FDG was injected intravenously via the ear vein. Static images were acquired 45 min following tracer injection. PET images were obtained using a single static 15-min acquisition followed by CT imaging for attenuation correction and anatomical co-registration using the nanoScan PET/CT (Mediso Systems, USA), modified from previously described methods (Davis et al., 2009). PET-CT images were reconstructed and co-registered using VivoQuant 3.5 (InviCRO). 3D spherical volumes of interest were drawn to measure ^11^F-FDG-PET signal surrounding infected hardware and in an uninfected reference point (anteriorly to L4). SUVs were derived. Uptake into bone and soft tissue was determined based on an ROI surrounding the hardware and using CT-derived thresholds for bone [>700 Hounsfield units (HU)]. The implant was excluded from the images prior to analysis. SUVs were derived and ratios between bone and soft tissue calculated.

### *Ex vivo* CT imaging and analysis of lumbar spine

Rabbits were euthanized on post-operative day 56 and the spine was harvested from L4 to L7, including all four vertebrae and surrounding soft tissue. *Ex vivo* CT imaging (Mediso Systems, USA) was performed to assess bone remodeling and density. Two ROIs were drawn per vertebra and the percentage of dense bone volume (HU>3000) divided by the total bone volume (HU>700) are presented (Bibb et al., 2015). The implant was removed prior to imaging.

### Statistical analysis

Prism software (GraphPad, La Jolla, CA, USA) was used to analyze the data. Data are presented as mean±s.e.m. Bone-to-soft tissue SUV ratios and percentages of dense bone were compared by two-tailed Mann–Whitney test. *P*-values <0.05 were considered statistically significant.

## References

[DMM045385C1] Aleissa S., Parsons D., Grant J., Harder J. and Howard J. (2011). Deep wound infection following pediatric scoliosis surgery: incidence and analysis of risk factors. *Can. J. Surg.* 54, 263-269. 10.1503/cjs.008210 21658334 PMC3191901

[DMM045385C2] Beiner J. M., Grauer J., Kwon B. K. and Vaccaro A. R. (2003). Postoperative wound infections of the spine. *Neurosurg. Focus* 15, E14 10.3171/foc.2003.15.3.14 15347232

[DMM045385C3] Bernthal N. M., Stavrakis A. I., Billi F., Cho J. S., Kremen T. J., Simon S. I., Cheung A. L., Finerman G. A., Lieberman J. R., Adams J. S. et al. (2010). A mouse model of post-arthroplasty Staphylococcus aureus joint infection to evaluate in vivo the efficacy of antimicrobial implant coatings. *PLoS ONE* 5, e12580 10.1371/journal.pone.0012580 20830204 PMC2935351

[DMM045385C4] Bibb R., Eggbeer D. and Paterson A. (2015). *Medical modelling: The Application of Advanced Design and Rapid Prototyping Techniques in Medicine*. Amsterdam, Boston: Elsevier/WP, Woodhead Publishing is an imprint of Elsevier.

[DMM045385C5] Bonnick S. L. (2008). Monitoring changes in bone density. *Womens Health (Lond.)* 4, 89-97. 10.2217/17455057.4.1.89 19072454

[DMM045385C6] Censullo A. and Vijayan T. (2017). Using nuclear medicine imaging wisely in diagnosing infectious diseases. *Open Forum Infect. Dis.* 4, ofx011 10.1093/ofid/ofx011 28480283 PMC5414026

[DMM045385C7] Chahoud J., Kanafani Z. and Kanj S. S. (2014). Surgical site infections following spine surgery: eliminating the controversies in the diagnosis. *Front. Med. (Lausanne)* 1, 7 10.3389/fmed.2014.00007 25705620 PMC4335387

[DMM045385C8] Cho O.-H., Bae I.-G., Moon S. M., Park S. Y., Kwak Y. G., Kim B.-N., Yu S. N., Jeon M. H., Kim T., Choo E. J. et al. (2018). Therapeutic outcome of spinal implant infections caused by Staphylococcus aureus: a retrospective observational study. *Medicine (Baltimore)* 97, e12629 10.1097/MD.0000000000012629 30290637 PMC6200525

[DMM045385C9] Davis S. L., Nuermberger E. L., Um P. K., Vidal C., Jedynak B., Pomper M. G., Bishai W. R. and Jain S. K. (2009). Noninvasive pulmonary [18F]-2-fluoro-deoxy-D-glucose positron emission tomography correlates with bactericidal activity of tuberculosis drug treatment. *Antimicrob. Agents Chemother.* 53, 4879-4884. 10.1128/AAC.00789-09 19738022 PMC2772305

[DMM045385C10] Dworsky E. M., Hegde V., Loftin A. H., Richman S., Hu Y., Lord E., Francis K. P., Miller L. S., Wang J. C., Scaduto A. et al. (2017). Novel in vivo mouse model of implant related spine infection. *J. Orthop. Res.* 35, 193-199. 10.1002/jor.23273 27116085 PMC5268448

[DMM045385C11] Gemmel F., Rijk P. C., Collins J. M. P., Parlevliet T., Stumpe K. D. and Palestro C. J. (2010). Expanding role of 18F-fluoro-D-deoxyglucose PET and PET/CT in spinal infections. *Eur. Spine J.* 19, 540-551. 10.1007/s00586-009-1251-y 20052505 PMC2899827

[DMM045385C12] Gordon O., Ruiz-Bedoya C. A., Ordonez A. A., Tucker E. W. and Jain S. K. (2019). Molecular imaging: a novel tool to visualize pathogenesis of infections in situ. *mBio* 10, e00317-19 10.1128/mbio.00317-19 31662452 PMC6819656

[DMM045385C13] Guiboux J.-P., Ahlgren B., Patti J. E., Bernhard M., Zervos M. and Herkowitz H. N. (1998). The role of prophylactic antibiotics in spinal instrumentation. A rabbit model. *Spine* 23, 653-656. 10.1097/00007632-199803150-00002 9549786

[DMM045385C14] Hammoud D. A. (2016). Molecular imaging of inflammation: current status. *J. Nucl. Med.* 57, 1161-1165. 10.2967/jnumed.115.161182 27173159 PMC5572792

[DMM045385C15] Hazer D. B., Sakar M., Dere Y., Altinkanat G., Ziyal M. I. and Hazer B. (2016). Antimicrobial effect of polymer-based silver nanoparticle coated pedicle screws: experimental research on biofilm inhibition in rabbits. *Spine* 41, E323-E329. 10.1097/BRS.0000000000001223 26571170

[DMM045385C16] Hegde V., Park H. Y., Dworsky E., Zoller S. D., Xi W., Johansen D. O., Loftin A. H., Hamad C. D., Segura T. and Bernthal N. M. (2018). The use of a novel antimicrobial implant coating in vivo to prevent spinal implant infection. *Spine* 45, E305-E311. 10.1097/BRS.0000000000003279 31593059

[DMM045385C17] Hu Y., Hegde V., Johansen D., Loftin A. H., Dworsky E., Zoller S. D., Park H. Y., Hamad C. D., Nelson G. E., Francis K. P. et al. (2017). Combinatory antibiotic therapy increases rate of bacterial kill but not final outcome in a novel mouse model of Staphylococcus aureus spinal implant infection. *PLoS ONE* 12, e0173019 10.1371/journal.pone.0173019 28245229 PMC5330510

[DMM045385C18] Jiménez-Mejías M. E., de Dios Colmenero J., Sánchez-Lora F. J., Palomino-Nicás J., Reguera J. M., Garcia de la Heras J., García-Ordonez M. A. and Pachon J. (1999). Postoperative spondylodiskitis: etiology, clinical findings, prognosis, and comparison with nonoperative pyogenic spondylodiskitis. *Clin. Infect. Dis.* 29, 339-345. 10.1086/520212 10476739

[DMM045385C19] Kouijzer I. J. E., Scheper H., de Rooy J. W. J., Bloem J. L., Janssen M. J. R., van den Hoven L., Hosman A. J. F., Visser L. G., Oyen W. J. G., Bleeker-Rovers C. P. et al. (2018). The diagnostic value of ^18^F-FDG-PET/CT and MRI in suspected vertebral osteomyelitis-a prospective study. *Eur. J. Nucl. Med. Mol. Imaging* 45, 798-805. 10.1007/s00259-017-3912-0 29256136 PMC5978906

[DMM045385C20] Laratta J. L., Shillingford J. N., Hardy N., Lehman R. A., Lenke L. G. and Riew K. D. (2017a). A dose-response curve for a gram-negative spinal implant infection model in rabbits. *Spine* 42, E1225-E1230. 10.1097/BRS.0000000000002205 28441310

[DMM045385C21] Laratta J. L., Shillingford J. N., Hardy N., Lombardi J. M., Saifi C., Romanov A., Lenke L. G., Lehman R. A. and Riew K. D. (2017b). Intrawound tobramycin powder eradicates surgical wound contamination: an in vivo rabbit study. *Spine* 42, E1393-E1397. 10.1097/BRS.0000000000002187 28399544

[DMM045385C22] Lin P. L., Maiello P., Gideon H. P., Coleman M. T., Cadena A. M., Rodgers M. A., Gregg R., O'Malley M., Tomko J., Fillmore D. et al. (2016). PET CT identifies reactivation risk in cynomolgus macaques with latent M*.* Tuberculosis. *PLoS Pathog.* 12, e1005739 10.1371/journal.ppat.1005739 27379816 PMC4933353

[DMM045385C23] Mansour A., Nabos J. F. and Nabos R. F. (1979). Psoas abscess: thirty-four years after pyogenic osteomyelitis of the spine. *Orthopedics* 2, 262-264. 10.3928/0147-7447-19790501-09 24822908

[DMM045385C24] McDermott H., Bolger C. and Humphreys H. (2012). Postprocedural discitis of the vertebral spine: challenges in diagnosis, treatment and prevention. *J. Hosp. Infect.* 82, 152-157. 10.1016/j.jhin.2012.07.009 22926135

[DMM045385C25] McHenry M. C., Easley K. A. and Locker G. A. (2002). Vertebral osteomyelitis: long-term outcome for 253 patients from 7 Cleveland-area hospitals. *Clin. Infect. Dis.* 34, 1342-1350. 10.1086/340102 11981730

[DMM045385C26] Miller R. J., Thompson J. M., Zheng J., Marchitto M. C., Archer N. K., Pinsker B. L., Ortines R. V., Jiang X., Martin R. A., Brown I. D. et al. (2019). In vivo bioluminescence imaging in a rabbit model of orthopaedic implant-associated infection to monitor efficacy of an antibiotic-releasing coating. *J. Bone Joint Surg. Am.* 101, e12 10.2106/JBJS.18.00425 30801375 PMC6738548

[DMM045385C39] Council National Research (US) Care Committee for the Update of the Guide for the and Animals Use of Laboratory (2011). *Guide for the Care and Use of Laboratory Animals*, 8th edn. Washington (DC): National Academies Press (US).

[DMM045385C27] Ofluoglu E. A., Zileli M., Aydin D., Baris Y. S., Kuçukbasmaci O., Gonullu N., Ofluoglu O. and Toplamaoglu H. (2007). Implant-related infection model in rat spine. *Arch. Orthop. Trauma Surg.* 127, 391-396. 10.1007/s00402-007-0365-0 17522873

[DMM045385C28] Ordonez A. A., Sellmyer M. A., Gowrishankar G., Ruiz-Bedoya C. A., Tucker E. W., Palestro C. J., Hammoud D. A. and Jain S. K. (2019). Molecular imaging of bacterial infections: overcoming the barriers to clinical translation. *Sci. Transl. Med.* 11, eaax8251 10.1126/scitranslmed.aax8251 31484790 PMC6743081

[DMM045385C29] Plaut R. D., Mocca C. P., Prabhakara R., Merkel T. J. and Stibitz S. (2013). Stably luminescent Staphylococcus aureus clinical strains for use in bioluminescent imaging. *PLoS ONE* 8, e59232 10.1371/journal.pone.0059232 23555002 PMC3595258

[DMM045385C30] Poelstra K. A., Barekzi N. A., Grainger D. W., Gristina A. G. and Schuler T. C. (2000). A novel spinal implant infection model in rabbits. *Spine* 25, 406-410. 10.1097/00007632-200002150-00003 10707383

[DMM045385C31] Pribaz J. R., Bernthal N. M., Billi F., Cho J. S., Ramos R. I., Guo Y., Cheung A. L., Francis K. P. and Miller L. S. (2012). Mouse model of chronic post-arthroplasty infection: noninvasive in vivo bioluminescence imaging to monitor bacterial burden for long-term study. *J. Orthop. Res.* 30, 335-340. 10.1002/jor.21519 21837686 PMC3217109

[DMM045385C32] Pull ter Gunne A. F., Mohamed A. S., Skolasky R. L., van Laarhoven C. J. H. M. and Cohen D. B. (2010). The presentation, incidence, etiology, and treatment of surgical site infections after spinal surgery. *Spine* 35, 1323-1328. 10.1097/BRS.0b013e3181bcde61 20150831

[DMM045385C33] Sampedro M. F., Huddleston P. M., Piper K. E., Karau M. J., Dekutoski M. B., Yaszemski M. J., Currier B. L., Mandrekar J. N., Osmon D. R., McDowell A. et al. (2010). A biofilm approach to detect bacteria on removed spinal implants. *Spine* 35, 1218-1224. 10.1097/BRS.0b013e3181c3b2f3 20445479

[DMM045385C34] Smith J. S., Shaffrey C. I., Sansur C. A., Berven S. H., Fu K.-M. G., Broadstone P. A., Choma T. J., Goytan M. J., Noordeen H. H., Knapp D. R. Jr. et al. (2011). Rates of infection after spine surgery based on 108,419 procedures: a report from the Scoliosis research society morbidity and mortality committee. *Spine* 36, 556-563. 10.1097/BRS.0b013e3181eadd41 21192288

[DMM045385C35] Sponseller P. D., LaPorte D. M., Hungerford M. W., Eck K., Bridwell K. H. and Lenke L. G. (2000). Deep wound infections after neuromuscular scoliosis surgery: a multicenter study of risk factors and treatment outcomes. *Spine* 25, 2461-2466. 10.1097/00007632-200010010-00007 11013497

[DMM045385C36] Sponseller P. D., Jain A., Shah S. A., Samdani A., Yaszay B., Newton P. O., Thaxton L.-M., Bastrom T. P. and Marks M. C. (2013). Deep wound infections after spinal fusion in children with cerebral palsy: a prospective cohort study. *Spine* 38, 2023-2027. 10.1097/BRS.0b013e3182a83e59 23963019

[DMM045385C37] Stavrakis A. I., Loftin A. H., Lord E. L., Hu Y., Manegold J. E., Dworsky E. M., Scaduto A. A. and Bernthal N. M. (2015). Current animal models of postoperative spine infection and potential future advances. *Front. Med. (Lausanne)* 2, 34 10.3389/fmed.2015.00034 26131448 PMC4469114

[DMM045385C38] Zoller S. D., Park H. Y., Olafsen T., Zamilpa C., Burke Z. D. C., Blumstein G., Sheppard W. L., Hamad C. D., Hori K. R., Tseng J.-C. et al. (2019). Multimodal imaging guides surgical management in a preclinical spinal implant infection model. *JCI Insight* 4, e124813 10.1172/jci.insight.124813 PMC6413782 30728332

